# Smartwatch-Based Eating Detection: Data Selection for Machine Learning from Imbalanced Data with Imperfect Labels

**DOI:** 10.3390/s21051902

**Published:** 2021-03-09

**Authors:** Simon Stankoski, Marko Jordan, Hristijan Gjoreski, Mitja Luštrek

**Affiliations:** 1 Department of Intelligent Systems, Jožef Stefan Institute, 1000 Ljubljana, Slovenia; markojordan1905@gmail.com (M.J.); mitja.lustrek@ijs.si (M.L.); 2 Jožef Stefan International Postgraduate School, 1000 Ljubljana, Slovenia; 3 Faculty of Electrical Engineering and Information Technologies, Ss. Cyril and Methodius University, 1000 Skopje, North Macedonia; hristijang@feit.ukim.edu.mk

**Keywords:** activity recognition, automated dietary assessment, smartwatch, inertial sensors, data selection, information fusion

## Abstract

Understanding people’s eating habits plays a crucial role in interventions promoting a healthy lifestyle. This requires objective measurement of the time at which a meal takes place, the duration of the meal, and what the individual eats. Smartwatches and similar wrist-worn devices are an emerging technology that offers the possibility of practical and real-time eating monitoring in an unobtrusive, accessible, and affordable way. To this end, we present a novel approach for the detection of eating segments with a wrist-worn device and fusion of deep and classical machine learning. It integrates a novel data selection method to create the training dataset, and a method that incorporates knowledge from raw and virtual sensor modalities for training with highly imbalanced datasets. The proposed method was evaluated using data from 12 subjects recorded in the wild, without any restriction about the type of meals that could be consumed, the cutlery used for the meal, or the location where the meal took place. The recordings consist of data from accelerometer and gyroscope sensors. The experiments show that our method for detection of eating segments achieves precision of 0.85, recall of 0.81, and F1-score of 0.82 in a person-independent manner. The results obtained in this study indicate that reliable eating detection using in the wild recorded data is possible with the use of wearable sensors on the wrist.

## 1. Introduction

Understanding people’s eating habits plays a crucial role in interventions promoting a healthy lifestyle. Obesity, which is a consequence of poor eating habits and increased energy intake, can be a major cause of cardiovascular disease, diabetes, or hypertension. Recent data show that the prevalence of obesity has increased significantly over the last three decades [1]. In 2015 over 600 million adults (13% of the total adult population) were classified as obese [2]. Additionally, in the European region, the prevalence of obesity is estimated to be 23%. In addition, in 2017, it was reported that poor diet had contributed to 11 million deaths globally [3]. Monitoring the eating habits of overweight people is an essential step towards improving nutritional habits and weight management. Another group of people who are in need of monitoring their eating habits are people with mild cognitive impairment and dementia [4]. They often forget whether they have eaten before and as a result eat lunch or dinner several times a day or not at all. Proper treatment of these problems requires an objective measurement of the time at which a meal takes place, the duration of the meal, and what the individual eats. This was our main motivation for developing a method for eating detection. Nevertheless, detection of eating is relevant for healthy people to coach them on nutrition so that they keep (or improve) their health [5].

The most commonly used tools for assessing eating behavior are meal recalls [6], food diaries [7], and food frequency questionnaires [8]. Unfortunately, these approaches to self-reporting are highly dependent on the memory of the users, which can lead to under and over-reporting of food intake [9,10]. Automatic and unobtrusive monitoring tools that can minimize these limitations are critical to identify temporal patterns of food and nutrient intake accurately in order to suggest interventions for a healthy lifestyle.

This topic has been intensively investigated by the research community over the last decade. Early research efforts in this field experimented with several types of sensors attached to different parts of the body [11,12,13,14,15,16,17]. Over time, these efforts have shortened the list of sensor types and positions, focusing on two main criteria: The ability of the sensors to capture patterns of eating and the practical applicability, which includes user comfort and acceptance. Furthermore, these sensors should be suitable for continuous wearing in a real-world setting for a long time. Several studies related to this problem show that combining data collected from different types of wearable devices with machine learning (ML) algorithms could be used to extract meaningful information about a person’s eating behavior. Although remarkable progress has been made, most of the systems are obtrusive, or based on assumptions that are not applicable in real-life conditions.

In this study, we focus on developing a practical solution for detecting when an individual is performing an eating activity using a non-invasive smartwatch. In particular, we propose a method for eating segments recognition using fusion of deep learning (DL) and ML algorithms. The following scientific contributions are made:

1. A novel ML approach for eating detection using smartwatch, which is robust enough to be used in the wild.
  - The approach incorporates virtual sensor streams extracted from DL models that recognize food-intake gestures. This step enables us to transfer knowledge from data with precisely labelled food intake gestures to our dataset.
  - To deal with unpredictable nature of data collected in the wild, the approach uses a novel two-step data selection procedure. The first step automatically cleans the eating class from non-eating instances. The second step selects representative non-eating instances that are difficult to distinguish and includes them in the training set.
2. A publicly available annotated dataset recorded in the wild without any limitations about the performed activities, meals, or cutlery. The duration of the collected data is 481 h and 10 min and it is collected using off-the-shelf smartwatch providing 3-axis accelerometer and gyroscope.
3. An extensive evaluation of the proposed method is carried out, including: (i) A step-by-step evaluation of each part proposed in the method; (ii) a comparison of the method with and without our proposed approach for data selection; (iii) a comparison between our approach and established methods for highly imbalanced problems; (iv) an analysis of the effects of training personalized models; (v) a comparison of the results obtained using feature sets from different combinations of modalities; (vi) an analysis of the results obtained using different types of cutlery for the recorded meals.

The paper is organized as follows: Section 2 gives an overview of the current state-of-the-art approaches for detection of eating activities using different types of wearable sensors, especially smartwatches that work with ML methods. In Section 3, we present the details of the collected dataset. In Section 4, each step of our proposed ML based method for eating detection is presented. Section 5 describes the experimental setup used in the study. The evaluation results are presented and discussed in Section 6. The paper is concluded in Section 7.

## 2. Related Work

Over the last decade, a number of wearable sensors for automating eating detection have been proposed and studied. As a result, the field of research has expanded rapidly, leading to different definitions of the problem. Some of the studies detected food intake gestures, while others detected eating activity. Additionally, many studies defined their problem as detection of meals. In addition, studies in this field have tried to find novelty and improvements by using new sensors. Regarding the placement of the sensors, the researchers mainly studied the neck, head, ear, and wrist. For each body location, they proposed approaches using devices with different detection modalities, such as acoustic [12,18,19], inertial [20,21,22], visual [16], EGG (electroglottography) [14], and similar.

Acoustic sensors were most commonly used to detect chewing and swallowing sounds, with devices attached to the neck and head. Sazonov et al. [17] proposed a method for swallowing detection based on a sound coming from a throat microphone placed over the laryngopharynx in the throat. Amft et al. [12] developed a chewing detection system using a condenser microphone embedded in an ear pad. Another study by Amft et al. [23] deals with an in-depth analysis of chewing sounds and specifies the methodology and the most appropriate position of the microphone (inner ear, directed towards the eardrum). Similarly, Bedri et al. [18] used ear-based device for detection of chewing instances on data recorded in real-life. Yatani and Truong [24] presented a wearable acoustic sensor attached to the user’s neck. Gao et al. [19] proposed to use off-the-shelf Bluetooth headsets to unobtrusively monitor and detect users’ eating episodes by analyzing the chewing sound using a deep learning classification technique.

Great efforts have also been made to develop an accurate method for eating detection using ECG and electromyography (EMG). Farooq et al. [13] proposed a test scheme to evaluate the validity of using EGG for food intake detection by placing a laryngograph around the participant’s neck during the experiment. Woda et al. [25] used EMG to investigate the influence of food hardness, bolus size, chewing cycles, and sequence duration on certain food types. Kohyama et al. [14] took into account the chewing effort of finely sliced foods using EMG. Zhang et al. [26] proposed a method using EMG sensors attached to eyeglasses.

More recently, studies have explored the possibility of detecting chewing segments and eating episodes using a proximity sensor placed on the neck [27,28], combined with a threshold-based algorithm. Similar to this, Zhang et al. [29] developed a multi-sensor necklace for detecting eating activities in free-living conditions. The combination of proximity, ambient light, and motion sensors show robust performance.

Although these approaches have shown promising results, there are privacy concerns, and very often the placement of the sensor affects the real-world practicality, due to discomfort and obtrusiveness. As a result, recent state-of-the-art methods focus on a shorter list of sensors embedded in unobtrusive mounted devices such as smartwatches and eyeglasses [30,31]. From the proposed devices for eating detection, wrist-worn devices stand out as the most practical and user friendly for real-world usage. This technology offers advantages in terms of detecting the timing and duration of eating activities in an unobtrusive, accessible, and affordable way, leading to a high level of acceptance of the technology [32].

The early studies done using data collected with wrist-mounted devices were mainly conducted in a laboratory setting [33,34,35,36]. These studies mostly focused on the detection of micro-level activities such as intake gestures [37,38,39]. For this purpose, they usually used objective ground-truth techniques such as recording with a video camera. The most commonly used ML algorithms in these studies are Decision tree [40,41,42,43], Hidden Markov Models [39,44,45], Support vector machines [30,46,47,48], and Random Forest [49,50,51]. Some of them also used a combination of algorithms [20,52]. The presented results show that these methods can accurately detect the number of intake gestures during a meal. However, they are not robust for usage in the wild due to the large number of gestures that could be mistaken as an intake gesture. As a result, recent studies started to include various non-eating activities in the laboratory setup to create more robust models that can work in the wild [22,53]. Mostly these are activities such as touching the face, combing the hair, brushing the teeth, and similar. Although these studies show remarkable results, non-eating gestures are numerous and varied, and it is difficult to replicate them naturally in controlled environments. This was shown in [54] where eating detection method tested in the wild failed to achieve the expected results. Consequently, the research field has rapidly expanded the testing of their detection models in the wild. This step resulted in significant differences in evaluation metrics (e.g., duration of meals, number of bites, etc.) between similar in-lab and in-the-wild studies [55].

The majority of studies that tested their method in the wild used training data recorded in a semi-controlled laboratory setting [22,56,57]. The main reason that these studies used laboratory data for training is that their method relies on detection of intake gestures for which precise labelling is required. For this purpose, most studies used a wearable camera or a static camera placed on the table where food is eaten.

Dong et al. [58] proposed a method for detecting eating moments using a data from a wrist-worn device. Their approach relies on the assumption that meals tend to be preceded and succeeded by periods of vigorous wrist motion. The data for this study were collected using a smartphone mounted on the wrist. Based on this, it is unclear if the placement and the weight of the phone affected the intake gestures movements. The proposed method is using expert features that focus mostly on the wrist rotational motion, which are later classified using a Naïve Bayes model. Even though this study showed great performance, their approach is not suitable for real-life usage due to the assumption that a period of increased wrist motion exists before and after every meal. An extension of their work [58] with data from 104 subjects showed more realistic results, achieving a sensitivity of 0.69 (from 0.81) and a specificity of 0.80 (from 0.82). Additionally, the authors stated that their initial hypothesis may not work in many different situations.

Thomaz et al. [22] investigated a method for inferring eating moments using data collected with a popular off-the-shelf smartwatch. For the training of their model, they used data collected in a semi-controlled laboratory setting. The proposed method recognizes each intake gesture separately and later the intake gestures are clustered within 60-min intervals. The evaluation of the method was done using data recorded in a real-life scenario. Their dataset contains recordings from seven subjects. Each subject recorded data for one day, documenting one meal per recording. Although there were not any explicit limitations about the dataset, we believe that the number of recordings is quite small to give a clear picture of how the model would perform in real-life situation. One drawback of the method is the requirement of precisely labeled intake gestures. The labelling procedure limits the training data to be collected in a laboratory setting because video recording of the meal is required.

Zhang et al. [21] proposed a method that uses advanced time-point fusion technique for detection of intake gestures. As a part of their method, they also developed a technique for clustering the false alarms into four categories in order to identify the main behaviors that are similar to intake gestures. They evaluated their method on a dataset recorded in the wild using a wearable video camera.

Recently, Kyritsis et al. [59] put forth an end-to-end Neural Network that detects food intake gestures. The neural network uses both convolutional and recurrent layers that are trained simultaneously. Next, they showed how the distribution of the detected intake gestures throughout the day can be used to estimate the start and end points of a meal. They evaluated their approach on a dataset recorded in a real-life scenario. Although their approach shows outstanding results, we find that the in the wild dataset used for the evaluation is quite limited, containing only six meals. Another problem with the dataset used is the limitation of cutlery. Only recordings where subjects ate their meals with a fork or spoon were included. We believe that this restriction is very strict because the dataset contains only a small fraction of the possible cutlery that could be used, which could lead to obtaining overly optimistic results. Moreover, the restriction on the cutlery used indirectly leads to a restriction on the possible meals that could be consumed.

In this study, we further expand our work done in [60], where we developed a method for detection of eating segments using data recorded completely in the wild, without any limitations regarding the consumed meals and performed activities. Our method works with labelled eating segments instead of precisely labelled intake gestures and it offers the possibility for easier recording of additional data. Such data can be used for fine tuning to a specific eating behavior. Moreover, the selection of features has proven to be effective in different fields [61,62]. Therefore, we employed a procedure to select most informative features and to reduce the complexity of the models. Furthermore, we propose a step for selection of training data that cleans the eating segments from non-eating periods as well as a step that selects non-eating instances that are difficult to distinguish and includes them in the training set.

## 3. Dataset

This section presents the dataset collected in the wild using a smartwatch. Previous work has shown that methods evaluated only with data recorded in laboratories give overly optimistic results and perform poorly when tested in the wild. In addition, previous studies show that eating styles vary greatly from person to person, suggesting that a sufficient number of meals from a multitude of participants are needed to develop a robust eating detection model.

In order to mitigate these limitations, we decided to design a specific data collection procedure. For this purpose, we recruited 12 subjects (10 males and 2 females). Mean age of the subjects was 29 ± 6 (range 20–41) and mean body mass index (BMI) was 23.2 ± 2 (range 19.7–27). Each subject wore a commercial smartwatch, Mobvoi TicWatch S, running on the WearOS operating system. For the data collection procedure, we developed an application that collects data from 3-axis accelerometer and 3-axis gyroscope. The collection procedure was performed with a sampling frequency of 100 Hz. Furthermore, we used self-reporting technique for obtaining the ground truth. For this purpose, the application also includes a button that is used to label the meal segment by simply pressing this button when the meal is started and again when the meal is finished. Additionally, the subjects were using an application on their smartphone, where they provided information about the type of the meal and the used cutlery. The participants were asked to wear the smartwatch on their dominant hand throughout the day until the battery is depleted. The recording procedure did not include any restriction about the type of meals that could be consumed, the cutlery used for the meal or the location where the meal took place.

**Figure 1:**
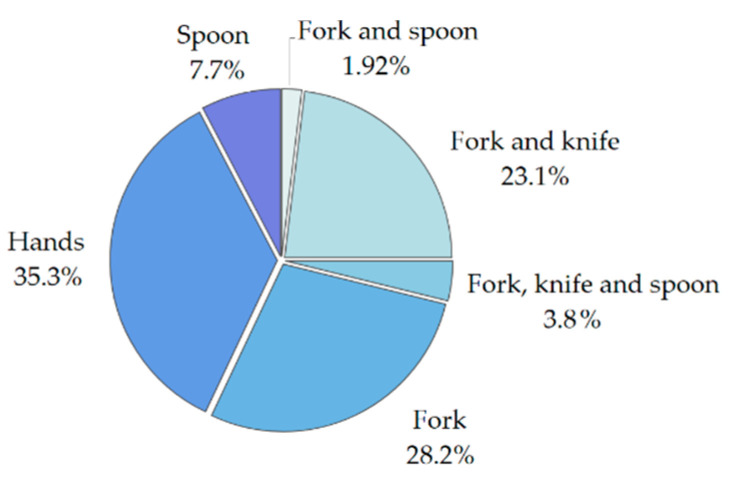
Distribution of the cutlery used for the recorded meals.

The total duration of the collected data is 481 h and 10 min, out of which 21 h and 42 min correspond to eating activities. Based on the information provided by the subjects, we also analyzed the different combinations of cutlery that were used during one meal. The distribution of the cutlery pieces used is shown in Figure 1. Hands refers to meals where no cutlery was used. Fork, knife, and spoon combination refers to meals where multiple dishes are eaten and the spoon is used separately from fork and knife.

## 4. Eating Detection Approach

**Figure 2:**
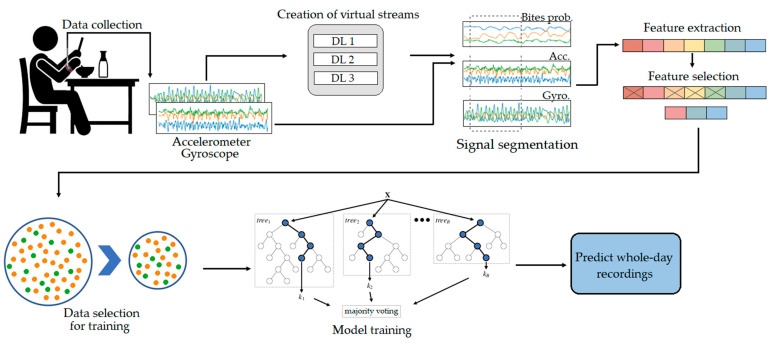
An overall pipeline of the proposed eating detection framework.

This section describes the approach for automatic detection of eating segments. The block diagram of the pipeline is shown in Figure 2. The approach is based on ML and consists the following steps: (i) Data preprocessing; (ii) virtual sensor stream extraction using DL; (iii) signal segmentation; (iv) feature extraction; (v) feature selection; (vi) training data selection; and (vii) two-stage ML model training. The first five steps (shown in the first row in Figure 2) are described in Section 4.1, while the remaining steps (shown in the second row in Figure 2) are described in Section 4.2.

### 4.1. From Input Data to Features

**Figure 3:**
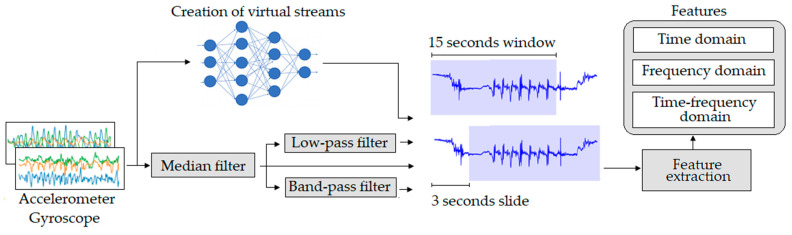
Raw data to features pipeline.

In this section, we describe the initial steps of our eating detection method. The preprocessing technique includes various filtering steps from which additional streams are extracted. In addition, we describe how we extract virtual streams from predictions of DL models. Finally, we describe in detail the features extracted from each stream used in the pipeline and the procedure for selecting only the most relevant ones. The steps described in this section are shown in Figure 3.

#### 4.1.1. Data Preprocessing and Segmentation

**Figure 4:**
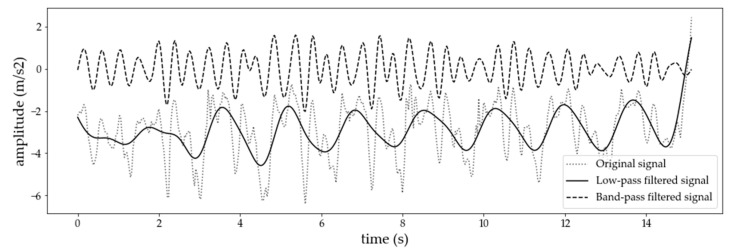
An example of an original and filtered (low-pass and band-pass) 15-s accelerometer x-axis signal.

The data collected for this study include signals from an inertial sensor with 6 degrees of freedom, i.e., three signals from an accelerometer and three signals from a gyroscope. The first step in the preprocessing pipeline involves interpolation of the signals to a fixed frequency of 100 Hz. This step was performed to handle inconsistencies in the sampling rate of the sensors. For simplicity, we worked with data sampled at 100 Hz in all experiments. However, similar studies [22,63] show that eating could be detected with data sampled at 25 Hz, which is important for practical implementation. Next, the signals were filtered using a fifth-order median filter. Moreover, the median-filtered accelerometer data were also processed with two more filters: Low-pass and band-pass. The output of this step is shown in Figure 4. The idea behind using two different types of filters (low-pass and band-pass) is to separate the gravitational force from the force caused from dynamic human motion. More specifically, by applying the low-pass filter, we keep only the gravitational force, while eliminating the force caused by dynamic movements. From the gravitational force we can extract useful information about the orientation of the sensor.

On the other hand, after applying the band-pass filter we only keep the medium frequency signal components, caused by dynamic human motion. They could provide useful information about the different hand gestures that are performed throughout the day. In fact, the signals were processed by a finite impulse response (FIR) low-pass filter with a cutoff frequency of 1 Hz, and a FIR band-pass filter with cutoff frequencies in the range of 5 to 10 Hz. For the gyroscope, we only used a low-pass filter in order to eliminate moments of highly dynamic human motion, since they are not related to eating activities. The characteristics of the filter used for the gyroscope are same as those for the accelerometer. Beside the raw sensor signals, we derived two virtual sensor streams that are useful for activity recognition tasks. We calculated the magnitude for both the accelerometer and gyroscope signals, which provide general information about the intensity of hand movement regardless of the direction of that movement.

The next step in the pipeline was to select the appropriate window size for the sliding-window segmentation technique. Previous studies related to eating detection typically used window sizes of up to 2 s, as their approach was based on precisely labelled intake gestures. However, our data contain only labelled eating segments. To increase the probability that an intake gesture is captured in a window, we used a longer window size. We determined the optimal window size empirically. The signals were segmented using a window size of 15 s with a 3-s slide between consecutive windows.

#### 4.1.2. Virtual Sensor Stream Extraction Using DL Models for Detection of Food Intake

We trained three DL models that are able to detect individual food intakes. The idea behind this step is to use the knowledge obtained from data where precisely labelled food intake gestures are present and to transfer it to our dataset. For this purpose, two publicly available datasets were used, Food Intake Cycle v1 (FIC) [64] and ISense [21]. The main reason we used two datasets is to create more robust models that could generalize well when they are used to produce predictions on our dataset. The FIC dataset consists of 21 sessions of 12 unique subjects, where each session corresponds to a recorded eating of a subject’s meal at the restaurant of a university, and the ISense dataset consist of recordings from 10 subjects. They were both recorded with different sampling frequencies, so we undersampled them to a 25 Hz frequency and fifth-order median filter was applied to slightly smooth the data. Both datasets contain labels of different micromovements that are part of the intake gesture. However, we merged these labels into two classes that show whether an intake gesture took place or not. These two classes were used for the training of the DL models. The models trained in such a way were used to generate predictions on our dataset, which does not contain labels for each food intake gesture separately. The output probabilities of the models that show whether food intake gesture has occurred were used as a virtual stream.

**Figure 5:**
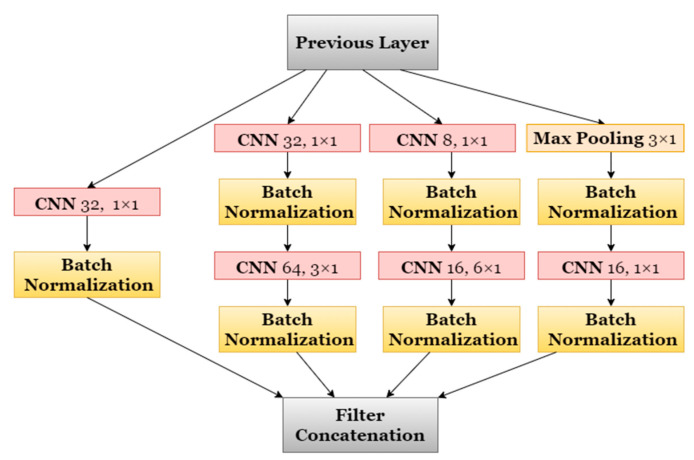
Architecture of Inception Block Type A.

The DL models to for the detection of individual food intakes also used sliding windows, like our main pipeline. Each window was input to three inception type [65] networks, which were chosen due to their remarkable achievements in related areas of DL. Two types of inception blocks were constructed. In Figure 5, the inception block of type A is shown. The inception block of type B is very similar to type A, except that it has twice the number of filters at each convolutional layer. The number of layers, together with the remaining parts of the architectures, were designed to keep the networks compact and easy to control.

**Figure 6:**
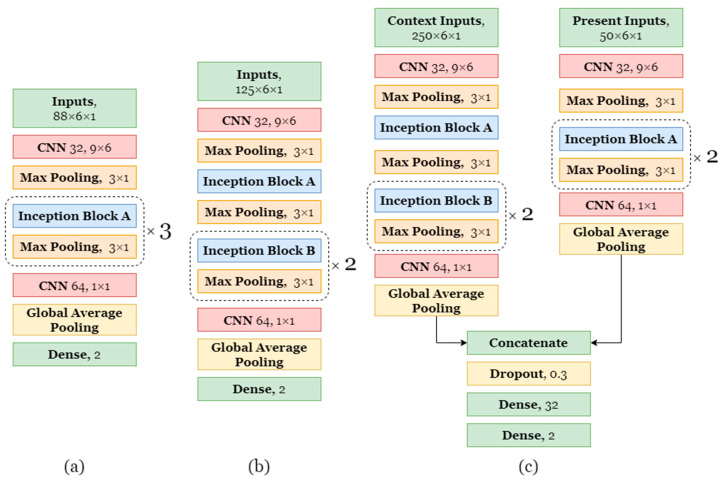
Architectures of the proposed models for bite detection. (**a**) Short architecture. (**b**) Medium architecture. (**c**) Long architecture.

In the inception blocks, the batch normalization [66] is applied after the convolutions. As activation function of the convolutional layers we used a rectified linear unit (ReLU) [67]. In every other convolutional or fully connected layer the activation is tanh. The exceptions are the last fully connected layers with two nodes, which have softmax. The selected architectures are shown in Figure 6. Instead of building a very large network with a very long training time, we rather chose three smaller ones. A RMSprop optimizer was used to optimize the weights of the network. The initial learning rate of the optimizer was set to be 0.001 and the rho value was 0.9. The batch size was set to 128. All of the 3 networks use the slide of 0.2 s for the sliding window approach, however the short model uses windows of size 3.5 s, the medium model of size 5 s, and the long model uses 10 s for the context window and 2 s for the present window. Context window consists of 4 s of the past, 2 s of present, and 4 s of future. Present data in the context window are identical to the data in the present one.

As a part of our food intakes detection method we tuned six hyperparameters. The first four determine what data are used for training, while the final two determine how the outputs of the models are interpreted. (i) Positivity threshold is the minimum percentage of samples inside a window labelled as intake gesture that are needed for this window to be considered a positive instance. (ii) Negativity threshold is the minimum percentage of samples not labelled as intake gesture that are needed for this window to be considered a negative instance. (iii) Bite length bound is the maximum length of an individual food intakes used for training; longer intakes are not used for training. (iv) Negative ratio represents the ratio between negative and positive instances in the training dataset, so it governs how negative instances are undersampled. (v) Bite probability threshold is the probability that has to be exceeded so that a window can be classified as a food intake. (vi) Bite distance is the minimum distance between two predicted individual food intakes. The first four hyperparameters are used for selection of the instances that are part of the training dataset. Once these hyperparameters are selected on the training dataset, a model is trained with them and predictions are made on the test dataset. During the hyperparameters optimization, the test dataset was not used. The optimal values obtained for each architecture are the following:

- Short architecture: Positivity threshold 0.36, negativity threshold 0.28, bite length bound threshold 5.5, negative ratio 5.
- Medium architecture: Positivity threshold 0.22, negativity threshold 0.28, bite length bound 6.5, negative ratio 5.
- Long architecture: Positivity threshold 0.30, negativity threshold 0.23, bite length bound 7, negative ratio 5.

The optimal values of all the hyperparameters per model were estimated on a constrained hyperparameter space, which we constructed based on expert knowledge. The optimization was done using the hyperopt [68] library, with the number of iterations set to 35.

#### 4.1.3. Feature Extraction

Features were calculated for seven different data streams, namely median, low-pass, and band-pass filtered accelerometer data, low-pass filtered gyroscope data, and the outputs from the three DL models. The features were designed to best describe the different aspects of each data stream. For example, the features extracted using the low-pass filtered accelerometer stream describe how the orientation of the device changes during different activities. On the other hand, the features extracted from the gyroscope stream provide information about the rotational movement of the wrist. In addition, the features extracted from the data streams obtained with the DL models attempt to capture different temporal structures of the recognized food intakes. In total, 2856 features were calculated and they can be roughly divided into three categories: Time-domain, frequency domain, and time–frequency features. The idea is to include multiple views of the signal from which the algorithm can choose the most informative features. More specifically, the time-domain features are used to distinguish persisting patterns or trends over time, while the frequency-domain features describe the periodicity of a signal over a range of frequencies. The time-frequency features are combination of the previous two groups and describe the changes of the frequencies over time.

**Time-domain features**: We extracted time-domain features that were used in our previous work and have proven to be effective in the AR domain [69]. In addition, we designed some eating-specific features that can better describe the eating gestures. These features can be divided into two categories, namely general time-series statistical features and domain expert features.

The statistical time-series features characterize the signal’s intensity and “shape”. In general, these features are not related to particular aspects of human gestures, postures, and movements. Instead they represent various ways to describe the information within the time-varying signals. The set of statistical features that were extracted directly from all sensor streams include the minimum, maximum, mean, median, standard deviation, variance, skewness, kurtosis, and similar.

In our previous work, inspired by the repetitive movement of the hand while eating, we designed a few features based on the auto-correlation. In particular, we explored the auto-correlation of the low-pass filtered accelerometer and gyroscope signals. These features were designed using the vector that is formed as the output from the auto-correlation function, which is calculated for multiple consecutive values. The final features are obtained with calculation of the following functions where the input is the previously formed vector: The number of peaks, number of zero-crossings, mean value of the distances between peaks, mean value of the distances between zero-crossings, and the area under the curve.

Furthermore, we extracted a few expert features that capture the wrist motion of the gestures. For these features a combination of the gyroscope and low-pass filtered accelerometer data streams was used. In particular, we calculated the mean and the variance of the roll and pitch values. These two metrics show the rotation along the x and y axis. Additionally, from the roll and pitch vectors we derived the following features: The number of peaks and total amplitude. We also calculated the amount of wrist roll motion, as well as the regularity of the wrist roll motion represented by percentage of time when the wrist is in roll motion.

The output of the DL models, which represents the probability that a food intake occurred, was treated as a time series for which we extracted features using the python package tsfresh. This package allows general-purpose time-series feature extraction. These features include the minimum, maximum, mean, variance, correlation between axes, their covariance, skewness, kurtosis, quartile values and the range between them, the number of times the signal is above/below its mean, and the signal’s mean change, among others. The same features were calculated for each data stream obtained from the output of the DL models.

**Frequency-domain features**: This group of features describe the periodicity of the signal and they were calculated using the power spectral density (PSD). The computation of the PSD is based on the fast Fourier transform (FFT). PSD estimates the power distribution of an input signal over a specific frequency range. The selected window size of 15 s is long enough to contain several intake gestures, which is required to extract useful information when calculating PSD of a signal. Based on our previous work [61], we calculated the following features from the PSD: five highest peaks of the PSD magnitude and their corresponding frequencies, energy that was calculated as the sum of the squared FFT component magnitudes, binned distribution, which is essentially the distribution of the FFT magnitudes into 10 equal sized bins ranging from 0 Hz to 20 Hz, and the skewness and kurtosis. These features were calculated for the accelerometer and gyroscope signals, as well as for the virtual streams produced from the DL models’ output.

**Time-frequency features:** We investigated another group of features based on a digital signal-processing technique called the continuous wavelet transform (CWT). This signal-processing technique allowed us to extract new features that could potentially provide information about the temporal dependencies of frequencies occurring in a signal. Although features based on the FFT have proven to be effective, the FFT decomposes a signal only in the frequency domain without any information about the time domain. CWT overcomes this limitation; more precisely, it has a high resolution in the frequency domain and also in the time domain, which allows us to know at which frequencies the signal oscillates and at what time these oscillations occur. A detailed description of CWT can be found in [70]. The Ricker wavelet was used as the mother wavelet. The computation of the CWT was performed for different values of the scaling and displacement arguments. The output values were taken directly as features.

#### 4.1.4. Feature Selection

The high number of data streams used for feature extraction resulted in a relatively high number of features. Consequently, we used a feature selection procedure to exclude those features that do not contribute to the performance of the models. In addition, using fewer features improves the computational efficiency of the proposed solution and simplifies its implementation. Furthermore, the development of a general model that is able to work in a person-independent manner should include a feature selection procedure to reduce the probability of overfitting to a particular person.

To begin with, we computed the mutual information (MI) between the features and the label. MI is a quantity that measures the reduction in uncertainty about the label given the knowledge of the feature. After that, we calculated the Pearson correlation coefficient for each pair of features. Once we have this information we start removing the redundant and uninformative features. If the correlation between a pair exceeded a threshold of 0.8 (strong correlation), we removed the feature with the lower MI.

A limitation of this feature selection procedure is its inability to deal with highly imbalanced problems [71]. The key features that are critical for the minority class may be lost if the feature selection is performed directly on imbalanced data. Therefore, before applying the feature selection procedure, we used a step in which we balanced the two classes. To obtain more heterogenous non-eating samples, each daily recording was balanced by a uniform selection of samples from the non-eating class. It is important to note that this balanced dataset was only used for the feature selection procedure.

The selection procedure was adjusted to work in a person-independent manner. For this purpose, we used the leave-one-subject-out (LOSO) technique with which we selected a feature set for each subject individually. This means that only the training data in each iteration was used for selecting features. Using the selected feature set in each iteration of the LOSO evaluation, predictions on the test subject were made.

### 4.2. Data Selection and Training of the ML Models

In this section, we describe our approach for selecting the most informative instances for training the ML models. Additionally, we present our model training procedure for detection of eating segments, which deals with the imbalanced nature of the problem.

#### 4.2.1. Data Selection Method

**Figure 7:**
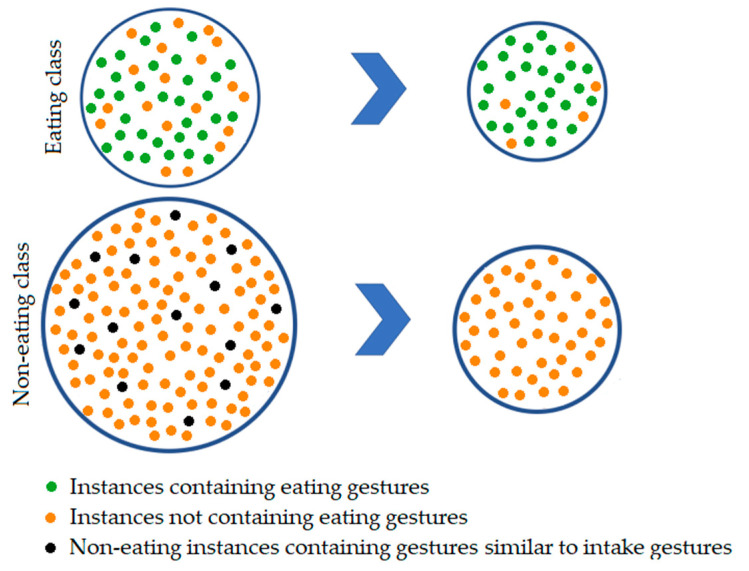
Composition of the eating and non-eating classes before and after all steps of the data selection procedure.

As already mentioned, our dataset contains labels for entire eating segments and not just for each individual food intake. The basic idea behind the data selection step is to drop parts of the eating segments that do not actually contain eating gestures. In [64], the authors used dataset that contains only precisely labelled food intakes, and they show that during a meal, on average only 40% of the time food intake gestures take place, while the other 60% are non-eating activities such as pauses between individual food intakes, conversations, use of a smartphone, and similar. In our dataset, the non-eating part consists of instances of which 90% contain gestures that are not similar to intake gestures and can be easily distinguished, and the remaining 10% contain gestures that are similar to food intake gestures. The composition of the eating and non-eating classes before and after the proposed data selection procedure is shown in Figure 7. To automatically drop longer segments between food intake gestures that are actually not related to eating, we developed a two-step data selection method and one final step for balancing both eating and non-editing classes.

1. The first step of the method cleans the non-eating segments. The idea is to eliminate those 10% of the instances that contain gestures that are similar to eating gestures. For this purpose, we used the EditedNearestNeighbors (ENN) method [72]. This method applies a nearest-neighbors algorithm and edits the dataset by removing the samples that do not agree enough with their neighborhood. For each sample in the non-eating class, the N nearest neighbors are computed using Euclidian distance, and if the selection criterion is not fulfilled, the sample is removed. The number of the nearest neighbors that are considered for the selection criterion is 5. The definition of the selection criterion requires that all nearest neighbors have to belong to the opposite class (eating) to drop the inspected sample from the non-eating class. The non-eating samples that do not contain gestures that are similar to eating gestures should not be greatly affected by the used selection criterion. Even though this assumption is a bit weak, the main reason that we rely on it is that the non-eating class is more numerous compared to the eating class, and excluding some non-eating samples, even if they are not very similar to eating samples, is not a problem.
2. After the first step of the undersampling technique, we expect that the non-eating class is comprised of instances that contain gestures that are not similar to eating gestures. The idea for the second step is to exclude instances from the eating class that do not contain eating gestures. For this purpose, we clean the eating class. Similar to the previous step, we again used ENN, with a small difference regarding the number of neighbors and the selection criterion. Here, we worked with the 7 nearest neighbors, and the majority vote of the neighbors is used to exclude a sample from the eating class. Due to the large number of non-eating samples that contain gestures that are not similar to eating, using the majority vote criterion most of the samples from the eating class that also do not contain gestures related to eating will be outvoted. Consequently, the eating class should mainly consist of samples that contain eating related gestures.
3. The last step of the data selection procedure is to create balanced training dataset. Usually, training a classifier on dataset with unbalanced classes results in poor performance. Therefore, for each daily recording, we undersampled the non-eating class, resulting in 60% non-eating and 40% eating instances. This was done using uniform undersampling of the non-eating class. By keeping more non-eating data, we intended to include more heterogeneous non-eating activities in the training set.

The data selection procedure was performed separately for each daily recording of a subject. The main reason for this was to reduce the search space for the nearest neighbors. Additionally, working together with data from several subjects can change the distribution of data for some subjects (e.g., the undersampled instances to be from only one subject). In addition, when using ENN, the similarity check was performed only with the most informative features selected with the method described in Section 4.1.4.

#### 4.2.2. Two-Stage Model Training

The proposed eating detection approach consists of two training stages. The first stage aims at training an eating detection models on an appropriate amount of representative eating and non-eating data, with a specific focus on problematic non-eating samples. The second stage takes temporal information into account and smooths the predictions. It is important to note that the generalization accuracy of the models was tested on the whole data of a subject and not just on the selected data that was used for training.

**Figure 8:**
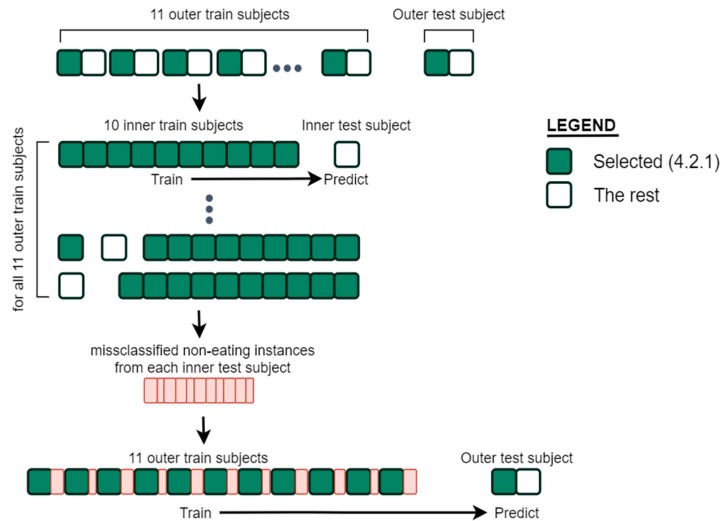
Model training procedure for one subject. The same procedure is repeated for each subject in the dataset.

The initial training data selected with our data selection method described in Section 4.2.1 contain only uniformly selected non-eating samples and it is shown with green squares in Figure 8. The regular approach would be to train a model on the balanced training dataset (selected in Section 4.2.1) and produce predictions for the whole data of a subject (green and white squares). However, this results in a large number of false positive predictions (non-eating recognized as eating). The main reason for this outcome is our inability to select eating-like activities and including them in the training data. Therefore, we developed a two-step training procedure that can select non-eating samples that are difficult for prediction and include them in the final training data for a particular subject. Only bursts of 7 or more consecutive misclassified non-eating samples were included in the initial training data, since adding all misclassified samples results in overfitting of the models. The number was selected experimentally.

The procedure for training and selecting misclassified samples was carried out as follows. The number of subjects in the dataset is denoted with N. For each of the N subjects in the dataset the same procedure was repeated. First, the whole data of a test subject (shown as outer test subject in Figure 8) are set aside. With the remaining N-1 subjects we perform the LOSO evaluation as described in Section 5. From the predictions made on each of N-1 inner test subjects in the LOSO evaluation, we select bursts of 7 or more consecutive misclassified non-eating instances. The new selected instances (shown with red squares in Figure 8) are different from those selected in Section 4.2.1. These instances are added to the training set, which consists of the selected instances (green squares) from N‑1 subjects. Finally, a model is trained on this set of data and predictions on the whole data from the subject that was left aside are made.

In the previously described procedure, the classification of the consecutive windows was independent. This means that the temporal information between the windows is not considered. However, in the second stage of the method, we postprocess the predictions by taking their temporal dependence into account. For instance, if a couple of consecutive windows are classified as “eating”, with only one window classified as “non-eating” in between, it is expected that the “non-eating” is a misclassification rather than a break between meals. To overcome this problem, we used a Hidden Markov Model (HMM) as additional model after the classification. In HMM, the actual activities are represented by the hidden states of the model, while the classified activities are represented by the emissions. The transition probabilities between the states and the probabilities of the observed emissions in each state are the parameters of the HMM. These parameters were calculated from the training data. The transition probabilities between the states were computed from the transition matrix of the real activities of the training set, i.e., the matrix of probabilities that one activity is followed by another. The probabilities of the observed emissions were calculated from the confusion matrix between the real and the predicted activities of the training subjects using an inner LOSO evaluation. The HMM smoothing was carried out using the Viterbi algorithm [73].

## 5. Experimental Setup

To estimate the performance of the proposed method, LOSO cross validation technique was used. With this technique, the initial dataset is split into N folds, where N is the number of subjects. This means that the models were trained on data from all subjects except for one on which we test the performance. The reported results were obtained from whole data predictions of a subject. The reason for this is to give a real picture of how good the developed method is in real-life settings.

Additionally, we decided to explore if the proposed method benefits from personalization. We evaluated the personalized models using a leave-one-recording-out (LORO) cross-validation technique. In other words, in the training dataset for each subject we included data from all other subjects, and all daily recordings from the subject except one, on which we later tested the performance of the trained model. The same procedure was repeated for each subject’s daily recording.

To assess the performance of the method on detecting eating moments, we used the following evaluation metrics: Recall, Precision, and F1-score. Each of the reported metrics was calculated using the eating activity as the positive class. The recall shows how many of the eating segments present in the test were detected as eating by the model, while the precision shows how many of the detected eating segments are in fact eating segments. The reported metrics reflect the ability of the models to detect eating moments at window level. Recall, Precision, and F1-score are calculated as shown in Equations (1)–(3):

(1) $$\mathrm{Recall}=\frac{\mathrm{TP}}{\mathrm{TP}+\mathrm{FN}}$$

(2) $$\mathrm{Precision}=\frac{\mathrm{TP}}{\mathrm{TP}+\mathrm{FP}}$$

(3) $$F1=\frac{2\cdot \mathrm{TP}}{2\cdot \mathrm{TP}+\mathrm{FP}+\mathrm{FN}}$$

where TP denotes true positives, TN denotes true negatives, FP denotes false positives, and FN denotes false negatives. In terms of eating detection, where the eating class is the positive class, these metrics can be described as follows:

- The TP value shows the number of windows from the eating class correctly classified as eating.
- The FP value shows the number of windows from the non-eating class classified as eating.
- The FN value shows the number of windows from the eating class classified as non-eating class.

## 6. Experimental Results

To explore the performance of our eating detection method, we carried out a series of experiments. In Section 6.1, we first present the results of the experiments done using the DL models. Section 6.2 shows the impact of each step included in our pipeline, as well as the final results. Next, in Section 6.3, evaluation of different comparison methods is presented. In Section 6.4, we present the method’s performance using feature sets from different modalities. In Section 6.5, we show the effect of personalization of our proposed methodology. Lastly, in Section 6.6, we present various analysis for each category of utensils that are present in the dataset.

### 6.1. Analysis of the DL Models for Food Intake Detection

Table 1 shows the performance of the DL models for food intake detection described in Section 4.1.2. The presented results are obtained from the both datasets that were used for training of the models. We can see that all three models perform similarly, achieving precision and recall around 0.75.

Given that these models could successfully learn the intake gestures characteristics from the dataset recorded in a laboratory setting, we conducted an experiment to see how well they would perform on our dataset recorded in the wild. One issue that arises when testing the models on our dataset is that it only contains labels for eating segments. Therefore, we included another step that postprocesses the detected individual food intakes and forms eating segments. The same postprocessing technique was used as described in Section 4.2.2. The obtained results are shown in Table 2. Even though the results shown here and the results from Table 1 are not directly comparable, in general we can see that the results are lower on our dataset. It can be also seen that a number of false positive gestures are detected, which means that the models could not distinguish very well gestures that are similar to those related to eating. However, this is expected if we have in mind that the models are trained on laboratory data in which only a limited number of non-eating gestures are included. It is important to note that the postprocessing step significantly reduced the false positive predictions, implying that the number of false positives generated by the DL models was initially even larger. In addition, the results suggest that the models failed to identify large number of intake gestures, which leads to less detected eating segments. We believe that the main reason for this is the limitation of the types of meals consumed, as well as the type of cutlery used in the training recordings. Nevertheless, the results show that the models are able to recognize eating in the wild to some extent, which we consider acceptable for transferring that knowledge and developing a more robust model on our dataset.

If we compare the performance of the models, we can see that the second model is the most balanced in terms of precision and recall. However, based on our analysis we observed that each model is able to capture a different aspect of eating. Therefore, we decided that a combination of all three models could help to detect eating more accurately. As a result, the output probability from all three models was used as a virtual stream in our proposed method.

### 6.2. Step-by-Step Evaluation of the Proposed Method

In this section, we conducted a detailed analysis of the proposed methodology to show the impact of each step used in the pipeline. Table 3 gives a complete picture of the results obtained in the conducted experiments. We analyzed the steps proposed in Section 4.2. In addition, we compare the same approach with and without the data selection method described in Section 4.2.1.

Row-wise comparison of the used evaluation metrics revels the improvements introduced at each of the steps. It also justifies the need to include several steps in our pipeline. In addition, the column-by-column comparison shows how our data selection methodology affects the performance of the models at each step.

- **First step:** The first row shows the results obtained using only balanced dataset for the training, without post-processing of the predictions. For those experiments where the data selection step is not used, only the classes are balanced. On the other hand, when data selection is used, as described in Section 4.2.1, the eating segments are undersampled and then we balance the eating and non-eating classes. The results show that the precision for both approaches, with and without the data selection step, is relatively low. This indicates that the method cannot accurately distinguish between activities similar to eating. However, the precision of the approach without data selection is higher compared to the approach where we used data selection. When the data selection step is used the non-eating instances that contain gestures similar to eating are excluded from the training and as a result the models detect them as eating instances.
- **First step + HMM:** The second row of the table shows the results after smoothing the predictions made in the first stage. Here, both the precision and the recall are significantly improved for both approaches. However, the precision value is again relatively low, indicating that further improvements are needed. The improvement in precision introduced by the smoothing suggests that probably only the short bursts of false positive predictions have been removed. Hence, we developed the second step training, which we expected to deal with this problem.
- **Second step:** The third row presents the results achieved with our proposed method in Section 4.2.2, excluding the post-processing part performed with HMM. Here our approach uses additional misclassified non-eating instances for training. As a result of this step, we can see that when using data selection, we get an improvement of 0.43 in precision, while the recall decreases by only 0.18. The results show that the second step solves the problem we have in the first step where many false positives are produced. Even though the recall value in the second step is lower when data selection is used, the f1-score, which is interpreted as a weighted average of precision and recall, shows that our method with the data selection step outperforms the same method without the data selection step by 0.03. The explanation for lower recall is that the models do not overfit to the eating class and only those parts of the meal that are related to eating are detected.
- **Second step + HMM:** The last row shows the results obtained after smoothing the predictions made in the second step. Again, the smoothing improved the results remarkably. For the approach where data selection was used, we can see that the precision is improved by 0.43 if we compare it with the second row of the table, while the recall only decreased by 0.07. This suggests that selecting and training on non-eating instances that are problematic for classification can significantly reduce the number of false-positive predictions, at the expense of a 0.07 reduction in recall, which we find acceptable. Furthermore, the comparison of the f1-score between the approach including data selection and the approach without data selection shows that the former is better by 0.07.

### 6.3. Comparison to Related Methods for Imbalanced Problems

In this section, a comparison with different algorithms developed for highly imbalanced problems is shown. With this experiment, we want to compare our proposed approach for learning from highly imbalanced data with methods that are already established in this field. For comparison, we used three methods: Balanced Random Forest (BRF) [74], EasyEnsamble (EE) [75], and Balanced Bagging (BB) [76]. BRF trains a classifier in which each tree of the forest will be provided balanced bootstrap samples. Similarly, EE is an ensemble of AdaBoost learners trained on different balanced randomly selected samples. BB is a similar implementation of the ensemble method Bagging, which includes an additional step to balance the training set at fit time. It should be noted that the obtained results from each method are postprocessed using HMM. The results of this experiment are shown in Table 4.

It can be seen that all three methods achieved a relatively high recall. However, the precision is quite low, considering that the results shown also include post-processing of the predictions. Although these three methods are quite different, the way they deal with the class imbalance problem is similar. Since they are ensemble methods, balanced bootstrap samples are provided as input in each iteration. However, it is very unlikely that most iterations will include cases that contain gestures similar to eating, since they only represent a small part of the entire dataset. As a result, the trained models are not robust and produce many false-positive results for instances that have similar characteristics to those in the eating class. Our method mitigates this limitation by using an inner LOSO evaluation from which we select bursts of misclassified non-eating instances. In this way, we are sure that the training data contains some instances that are difficult to distinguish, and that they are likely to include gestures similar to eating. As a result, the results obtained with the proposed methodology show higher precision and recall.

### 6.4. Method’s Performance Using Feature Sets from Different Modalities

Table 5 shows the results obtained using feature sets from different combinations of modalities. Given the modalities available, the features were grouped into three categories, i.e., features extracted from the accelerometer, the gyroscope, and from the output of the DL models. We investigated the performance of the method using features from each modality individually as well as their combinations: accelerometer + gyroscope (AG), accelerometer + DL (AD), gyroscope + DL (GD) and accelerometer + gyroscope + DL (AGD). The comparison of the results using features from a single modality shows that those from the gyroscope are most informative. However, the combination of features from two modalities leads in all cases to better results compared to the results obtained using features from a single modality. In addition, the use of features from all three modalities leads to an even better classification performance than the use of features from two modalities. In fact, our idea to extract features from the output of the DL models and combine them with those of accelerometer and gyroscope gives new insights into the method, improving both precision and recall.

### 6.5. Personalized Models

The experiments we carried out showed that eating styles vary greatly from person to person. Therefore, we decided to investigate the effect of personalized models. It is generally known that personalized models improve the performance of activity recognition. In this experiment, the training dataset for a given subject consists of recordings from all other subjects and all daily recordings that the subject has recorded except one, which is used to test the performance of the trained model. Such personalization is valuable for real-life use because the subject can only record a few daily activities and meals that can later be used to fine-tune the eating detection model for their specific eating style.

**Figure 9:**
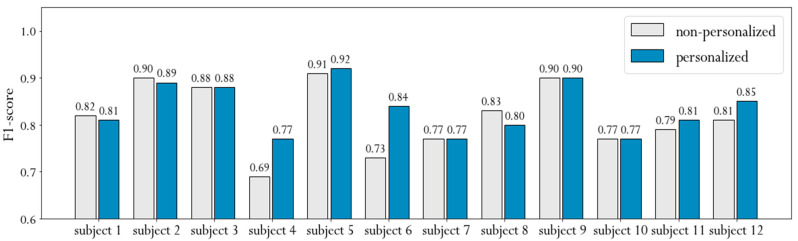
F1-score of personalized and non-personalized models shown for each subject separately. Non-personalized results achieved using LOSO evaluation, personalized results achieved using leave-one-recording-out (LORO) evaluation.

Figure 9 shows the f1-scores obtained from non-personalized and personalized models separately for each subject. The average f1-score of the non-personalized approach is 0.82, while the personalized approach achieves an average f1-score of 0.84. This could indicate that the method we propose does not benefit greatly from personalization. However, if we analyze the performance for each subject individually, we find that personalization of subjects 4 and 6 leads to an improvement of 0.08 and 0.11, respectively. Although the improvement is quite large, it is even more important that these two subjects have the lowest non-personalized results. This suggests that subjects with specific eating style can benefit greatly if we include personal recordings in the training dataset. Furthermore, it implies that our method can effectively use personal data in certain cases, even if only a small part of the whole training set is personal data.

### 6.6. Method’s Performance by Cutlery Type

**Figure 10:**
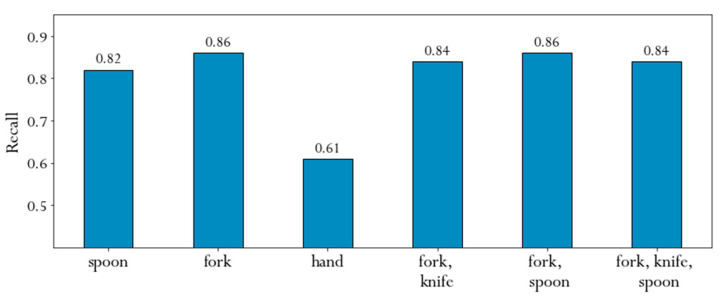
Average recognition for each type of cutlery.

In this section, we examined how well the proposed method could generalize to different types of cutlery used for the recorded meals. For each of the meals, the subjects provided information about the meal they consumed and whether or not they used cutlery. If they used cutlery, they also indicated the type of cutlery. Based on this information, we grouped the cutlery used into six groups, namely spoon, fork, hand, fork-knife, fork-spoon, and fork-knife-spoon. The distribution of the cutlery used for the meals is shown in Section 3. Figure 10 summarizes the performance for each group identified in terms of recall. We used this evaluation metric because it shows how many of the eating instances were actually identified as eating.

The figure shows that the eating was recognized well for all categories except the hand. Hand-eaten meals are not always eaten in the conventional way at a table. Very often people eat with their hands while walking or standing, which results in additional noise in the data. An interesting result is that with the proposed method, meals eaten with a fork and a knife can be successfully detected, although people eating with a fork and a knife at the same time usually perform the intake gesture with the non-dominant hand. This suggests that our method can learn the movement of the dominant hand when using a knife. 

## 7. Conclusions

In this study, we presented a novel approach for detection of eating segments with a wrist-worn device and fusion of ML and DL. We collected an annotated dataset recorded in the wild without any restrictions about the performed activities, meals, or utensils. The total duration of the collected data is 481 h and 10 min, out of which 21 h and 42 min correspond to eating activities. The data were collected using an off-the-shelf smartwatch providing 3-axis accelerometer and gyroscope data. The dataset is publicly available and we hope that it will serve researchers in future studies. Furthermore, we believe that this dataset could be used as a benchmark for testing various approaches for detecting eating segments in the wild.

The proposed framework for the detection of eating segments consists of two parts. First, we extract virtual sensor modalities using pre-trained DL models. For both raw and virtual sensor modalities, a comprehensive feature set is extracted, from which only the most relevant ones are selected using a feature selection algorithm. In the second part, we focused on selection of data for training, which is the main contribution of this study. For this purpose, we developed a data selection step that cleans the eating class from non-eating instances as well as a training step that selects non-eating instances that are difficult to distinguish and includes them in the training set.

The effectiveness of the individual steps of the proposed method was verified by a step-by-step evaluation. Our idea to train a model on instances that are difficult to distinguish leads to a better classification. Furthermore, the last step of the method shows that the recognition of eating segments can be significantly improved by incorporating temporal dependence between the individual recognitions. The experiments also show that the highest performance in the detection of eating segments is achieved when the model is trained on data processed with our proposed data selection method.

Overall, our eating detection framework achieved a precision of 0.85 and recall of 0.81, which show that the proposed method is capable of detecting eating segments throughout the day and is robust enough to cope with data from participants about whom it had no prior knowledge. Additionally, we would like to highlight the real-life evaluation as it shows the robustness of the method while dealing with many different activities that could be confused with eating, as well as identifying meals taken in many different environments while using different type of cutlery.

We did some additional analyses of the performance of the proposed method. The comparison with established methods for dealing with highly imbalanced problems shows that our method can better select the data on which the classifier is trained. Furthermore, analysis of the results obtained with feature sets from different combinations of modalities shows that our idea to extract features from the output of the deep learning models and combine them with those of accelerometer and gyroscope improves both precision and recall. Moreover, the comparison of the non-personalized and the personalized models shows that subjects with specific eating style can benefit greatly if we include personal recordings in the training dataset. This implies that our method can effectively use personal data in certain cases, even if only a small part of the whole training set is personal data.

For future work, we plan to incorporate contextual information alongside the sensor data from a smartwatch to eventually develop models of human eating behavior that can be used to provide adaptive and personalized interventions. Studies have repeatedly shown that context awareness plays an essential role in systems dealing with activity recognition [77]. Therefore, we plan to collect data about the location via GPS or wi-fi access points, which might help learning where the subjects usually have meals. As a part of this step, we plan to investigate various techniques for information fusion that have proven to be effective in different fields [78,79]. In addition, we plan to adjust the proposed method for real-time usage in order to assess different aspects of human eating behavior. Using such a method allows us to propose various real-time interventions that will focus on obesity preventions. At the moment, our method uses a small number of features that are selected using the feature selection algorithm. This means that the trained models are not very complex and the features could be extracted even with limited computational resources. However, if a very limited device is used, the DL models should be omitted. Furthermore, a smartwatch offers limited battery life, which does not allow such computations to be done frequently, so we need an optimized method that can make expensive computations only when it is critical for the eating detection. To achieve this, we plan to extend our previous work [80], where we developed an eating-specific trigger that activates the ML pipeline only when movements towards the head are detected.

## Figures and Tables

**Table 1:** Results of the DL models on the FIC and ISense datasets—food intake detection.

	FIC Dataset			ISense Dataset		
Method	Precision	Recall	F1-Score	Precision	Recall	F1-Score
Short architecture	0.73	0.82	0.77	0.68	0.78	0.72
Medium architecture	0.75	0.77	0.75	0.73	0.78	0.75
Long architecture	0.75	0.8	0.76	0.67	0.72	0.69

**Table 2:** Results of the deep learning (DL) models in combination with Hidden Markov Model (HMM) obtained from our dataset—eating segment detection.

Method	Precision	Recall	F1-Score
Short architecture + HMM	0.66	0.61	0.64
Medium architecture + HMM	0.69	0.66	0.67
Long architecture + HMM	0.75	0.56	0.63

**Table 3:** Average precision, recall, and f1-score for each step used in the proposed method, with or without data selection. Leave-one-subject-out (LOSO) evaluation. Hidden Markov Model (HMM).

Method	Without Data Selection			With Data Selection		
	Precision	Recall	F1-Score	Precision	Recall	F1-Score
1st step	0.47	0.79	0.57	0.33	0.82	0.46
1st step + HMM	0.52	0.85	0.64	0.42	0.88	0.55
2nd step	0.61	0.74	0.65	0.76	0.65	0.68
2nd step + HMM	0.7	0.85	0.75	0.85	0.81	0.82

**Table 4:** Average precision, recall, and f1-score achieved with machine learning (ML) methods for imbalanced data. LOSO evaluation. Balanced Random Forest (BRF), EasyEnsamble (EE), Balanced Bagging (BB), Hidden Markov Model (HMM).

Method	Precision	Recall	F1-Score
BRF [74] + HMM	0.41	0.9	0.54
BB [75] + HMM	0.52	0.89	0.64
EE [76] + HMM	0.38	0.92	0.53
Ours	0.85	0.81	0.82

**Table 5:** Average testing precision, recall, and f1-score achieved using feature sets from different combination of modalities. LOSO evaluation. A—accelerometer, G—gyroscope, D—deep learning output.

Metrics	Modality Combination						
	A	G	D	AG	AD	GD	AGD
Precision	0.72	0.77	0.78	0.79	0.82	0.84	0.85
Recall	0.79	0.74	0.68	0.8	0.8	0.77	0.81
F1-score	0.73	0.73	0.72	0.79	0.8	0.79	0.82

## Data Availability

The dataset used in this study can be found at: https://github.com/simon2706/EatingDetectionIJS (accessed on 6 March 2021).
